# GnRHa for Ovarian Protection and the Association between AMH and Ovarian Function during Adjuvant Chemotherapy for Breast Cancer

**DOI:** 10.7150/jca.31859

**Published:** 2019-07-10

**Authors:** Ying Zhong, Yan Lin, Xinqi Cheng, Xin Huang, Yidong Zhou, Feng Mao, Yajing Wang, Jinghong Guan, Songjie Shen, Yali Xu, Li Peng, Yan li, Xi Cao, Qiang Sun

**Affiliations:** 1 Department of Breast Disease, Peking Union Medical College Hospital, Shuaifuyuan, Wangfujing, Beijing 100730, China; 2 Department of Clinical laboratory, Peking Union Medical College Hospital, Shuaifuyuan, Wangfujing, Beijing 100730, China.

**Keywords:** Breast cancer, Chemotherapy, Ovarian function, GnRH agonist, AMH

## Abstract

**Background**: Chemotherapy impairs ovarian function in premenopausal breast cancer patients. Many breast cancer patients experience menopause earlier and therefore lose their reproductive abilities. The protective effect of gonadotropin-releasing hormone agonist (GnRha) upon the ovary is clearly apparent for hormone receptor (HR) negative patients, although the available data is not consistent for the patient body as a whole when considered regardless of HR status. It is also unknown whether levels of Anti-Mullerian Hormone (AMH) can reflect the influence of chemotherapy upon the ovary.

**Methods**: We randomly assigned 98 premenopausal breast cancer patients regardless HR-positive or -negative to receive either standard chemotherapy with GnRHa (GnRHa group) or standard chemotherapy without GnRHa (control group). Our primary end point was ovarian failure rate (OVF) at 1 year. In addition, we observed the change of AMH level during chemotherapy and the association between AMH and OVF.

**Results**: OVF was significantly lower (44.7%) in the GnRHa group than in the control group (80.6%; P=0.002). Median AMH levels were significantly higher before chemotherapy when compared to after 1/2cycles of chemotherapy, both in the GnRHa group (1.86ng/ml vs 0.12ng/ml; P=0.000) and in the control group (1.57ng/ml vs 0.10ng/ml; P=0.000). OVF was 91.3% in the AMH baseline level <1.1ng/ml group and 63.5% in the AMH baseline level >1.1ng/ml group (P=0.013).

**Conclusion**: Data showed that GnRHa may have a protective effect on young breast cancer patients regardless of HR during chemotherapy. AMH could reflect changes of OVF during chemotherapy and predict OVF after chemotherapy.

## Introduction

Approximately 6% of patients with breast cancer are less than 40 years old, and 1 in 200 people under 40 are likely to develop breast cancer^1^. The mean age of diagnosis for breast cancer patients in China is 45-55 years, which is far younger than Western women^2^. Young age has been shown to represent an independent predictor of poor prognosis in breast cancer^3^-^5^ and most patients in this age group receive chemotherapy. These patients have a high risk of transient or permanent amenorrhea, and there is more long-term risk of premature ovarian failure in patients who continue to undergo or recover their menstrual cycle^6^. A previous study estimated that one year of chemotherapy will lead to 1.5 years of reproductive loss^7^.

The onset of premature menopause depends on the age of the patient and the type of chemotherapy administered^8^. Premature ovarian failure has significant consequences, including infertility, sexual dysfunction and vasomotor symptoms^9^. Previous studies have shown that young survivors of breast cancer consider premature menopause, sexual dysfunction and infertility to represent the most distressing aspects of their cancer experience^10^. Due to concerns about infertility, 29% of breast cancer patients will request changes to the decisions made about their treatment 11 One previous study of breast cancer patients younger than 40 found that 68% of these patients discussed fertility problems with their doctors prior to the treatment commencing. Some of these patients refused chemotherapy or changed their chemotherapy regimen because of reproductive concerns, and 10% of patients took measures to protect their reproductive function^12^. Another study used a questionnaire to survey breast cancer patients younger than 35 years; 59% of patients expressed a desire to have more children and 8% of patients said they would not wish to receive chemotherapy because it could reduce their fertility^13^. Consequently, it is very important for young breast cancer patients to protect their ovarian function during chemotherapy. In addition, it is important for clinicians and patients to understand the state of ovarian function and to be able to predict the effect of chemotherapy on ovarian function. Thus far, no laboratory test has been able to accurately reflect ovarian function and predict ovarian function after chemotherapy.

Anti-Mullerian Hormone (AMH) is released from the granulosa cells of antral follicles and can be measured by serum concentrations, which are known to be proportional to the development of ovarian development. Consequently, AMH is considered to represent a marker of ovarian aging^14^. Levels of AMH provide an indirect indicator of the number of antral and pre-antral follicles in the ovary and are widely used in clinical practice^15^. Serum levels of AMH have also been shown to indirectly reflect the remaining primordial follicles of the ovarian reserve. Consequently, AMH can be used to predict reproductive longevity^16^, ^17^ and has become a biochemical marker of ovarian reserve^17^.

GnRHa can protect ovarian function during chemotherapy for hormone receptor (HR)-negative breast cancer patients^18^. However for HR-positive patients, the effect has not been clearly defined. This study aimed to examine whether GnRHa can protect ovarian function during chemotherapy, regardless of the state of the (HR), to investigate whether AMH levels can reflect changes of ovarian function during chemotherapy and finally, to investigate whether AMH levels can predict regular menstruation after chemotherapy.

## Materials and Methods

### Material

Detection of the levels of AMH, Follicle-Stimulating Hormone (FSH), Prolactin (PRL), Estradiol (E2), luteinizing hormone (LH), Testosterone (T) detection was conducted using an automated chemiluminescence immunoassay analyzer (Beckman Coulter UniCel DXI800, Brea, CA, USA) along with corresponding reagents, calibration materials and quality control materials.

### Patients

Premenopausal women, aged 18 to 45 years, were eligible for enrollment if they had operable stage I to IIIA breast cancer, regardless of hormone receptor status, for which treatment with adjuvant anthracyclines-containing chemotherapy was planned. The use of trastuzumab was permitted in patients with tumors which over-expressed human epidermal growth factor receptor 2 (HER2). Estrogen receptor (ER) and Progesterone receptor (PR) were performed at a clinical laboratory in PUMCH hospital by routine methods. All patients provided written informed consent for participation. Eligible participants were administered with tamoxifen if their hormone receptor status was positive. Patients were excluded for the following reasons: stage IV breast cancer or with distant metastasis; presence of other malignancies over the last 5 years; undergoing chemotherapy or receiving GnRHa, fitted with an intrauterine device, taking ovulation-promoting drugs or oral contraceptives within the previous three months; definite diagnosis of polycystic ovarian syndrome, irregular menstruation, amenorrhea; pregnancy or lactation.

### Study Design

In this study, patients were randomly assigned, in a 1:1 ratio, to standard adjuvant chemotherapy with the GnRH agonist goserelin (chemotherapy plus goserelin group) or to chemotherapy without goserelin (chemotherapy-alone group). The choice of the standard anthracyclines-containing chemotherapy regimen was left to the discretion of the clinical doctors. For patients randomly assigned to the chemotherapy plus goserelin group, we administered goserelin at a dose of 3.6 mg subcutaneously every 4 weeks from within 1 week of the initial chemotherapy dose and was continued to within 2 weeks of, or after, the final of chemotherapy.

Randomization was stratified according to age (<40 years *vs.* 40 to 45 years) and hormone receptor status (positive or negative). The primary objective was to investigate the rate of ovarian failure (OVF) which was defined as amenorrhea for the preceding 6 months. Patients who became pregnant were considered not to have had ovarian failure. Additional end points were disease free survival (DFS) and overall survival (OS). Events of overall survival included deaths due to any cause while events of disease free survival included breast cancer recurrence and metastasis. In a previous study^19^, ovarian reserve decline was defined as when AMH levels were lower than 1.1ng/ml; consequently, in the present study, we separated our patients into two groups according to their AMH levels (<1.1ng/ml and >1.1ng/ml) and then investigated the association between OVF and AMH grouping.

### Statistical Analysis

We originally aimed to recruit 240 eligible patients. We estimated that with this sample size, and using a two-group binomial design, our study would have more than 80% power to detect an absolute reduction of 15% points in the rate of ovarian failure, at a one-sided significance level of 0.025. Primary analysis was based on Chi-square tests and the Mann-Whitney test. In addition, we examined levels of AMH, FSH, E2, PRL and T before chemotherapy, 1/2 cycles after chemotherapy, 6 months after chemotherapy and 1 year after chemotherapy. We analyzed patient characteristics according to randomization group and stratification variables. We also observed the changes of AMH during and after chemotherapy using the Wilcoxon signed rank test and analyzed the relationship between clinical features including AMH and ovarian failure by Chi-square tests. Finally, exploratory Kaplan-Meier curves for disease free survival were calculated. According to the study-design specifications, a one-sided alpha level of 0.025 was used to indicate statistical significance for the primary end-point analysis of ovarian failure; for all other P values, a two-sided alpha level of 0.05 was used to indicate statistical significance. The cutoff date for all analyses was June 2017.

## Results

### Patients

We recruited and randomized a total of 98 patients between August 2015 and November 2016. One patient was not eligible and another patient was not evaluated up to the end point of this study. Consequently, our final analysis involved 96 patients (45 in the chemotherapy-alone group and 51 in the chemotherapy plus goserelin group). The median follow-up time was 15 months at the end of the analysis. Patient baseline characteristics and hormone levels are shown in Table 1 and Table 2. The median age of our patients was 39.0 years. All patients received anthracycline-based therapy and 79% of patients received therapy featuring either paclitaxel or docetaxel. The clinical characteristics of the two groups were well balanced. All hormone receptor positive patients received endocrine therapy, and the endocrine therapy drug was toremifen. There were no significant differences in the levels of AMH, FSH, LH, E2 or T between the two groups at baseline.

### AMH levels

There was a statistically significant relationship between patient age and AMH levels. Patients below and above the age of 40 were compared with regards to median AMH level (2.23ng/ml and 1.23ng/ml, respectively), median FSH level (5.91mIU/ml and 6.82mIU/ml, respectively), median E2 level (48.80pg/ml and 53.00pg/ml, respectively), median LH level (4.90mIU/ml and 5.27mIU/ml, respectively) and T (0.44ng/ml and 0.41ng/ml, respectively). Our analysis showed that the relationship between age and median AMH level was statistically significant (P=0.003; Table 2).

AMH level decreased significantly during chemotherapy in the chemotherapy plus goserelin group and in the chemotherapy-alone group. However, there was no significant difference, in terms of median AMH levels when compared before and 1 year after chemotherapy for either the chemotherapy plus goserelin group (P=0.141; Table 3) or in the chemotherapy-alone group (P=0.109; Table 3).

There was no significant difference in median AMH levels between the two groups when compared before chemotherapy (P=0.561; Mann-Whitney test; Table 1); in the chemotherapy-alone group, median AMH level before chemotherapy was 1.57 ng/mL, as compared to 1.86 ng/mL in the chemotherapy plus goserelin group. In the chemotherapy plus goserelin group, the AMH level after 1/2 cycles of chemotherapy was 0.12 ng/mL, which fell to 0.04 ng/ml after 6 months; both of these levels were significantly lower than the median AMH level prior to chemotherapy (1.86 ng/mL; p<0.05; Table 3). In the chemotherapy plus goserelin group, the median AMH level after 1 year of chemotherapy was 0.05 ng/ml, which was lower than the level of 1.86 ng/mL before chemotherapy but was not statistically significant (p>0.05; Wilcoxon Signed Ranks Test; Table 3).

In the chemotherapy-alone group, the median AMH level after 1/2 cycles of chemotherapy was 0.10 ng/mL, and 6 months after chemotherapy had fallen to 0.03; these levels were both significantly lower than the pre-chemotherapy level of 1.57 ng/mL, lowered as compared to 1.57 ng/mL (p<0.05; Wilcoxon Signed Ranks Test; Table 3). In the chemotherapy-alone group, median AMH level after 1 year of chemotherapy was 0.09 ng/mL, which was lower than the level before chemotherapy (1.57 ng/mL) but was not statistically different (p>0.05; Wilcoxon Signed Ranks Test; Table 3).

### Ovarian Dysfunction

Our study investigated four different factors which may be potentially associated with ovarian function recovery: age, baseline AMH, the administration of GnRHa and the addition of endocrine therapy (Table 4). Of the 96 patients, 74 valid information on their menstrual status was obtained. In 74 patients, 2 baseline blood samples were not detected valid information. In 74 patients, endocrine therapy was not available in one patient.

In terms of age, our analysis showed that younger women (<40 years) had a significantly lower rate of OVF (ovarian function failure) than older women (>=40 years) after treatment (P=0.002; Chi-square test; Table 4). Other analysis showed that detectable levels of baseline AMH (≥1.1 ng/ml) were significantly associated with a lower rate of OVF (P=0.013; Chi-square test; Table 4) and that the administration of GnRHa was significantly associated with a lower rate of OVF (P=0.002; Chi-square test; Table 4). Furthermore, OVF tended to be higher in patients receiving to endocrine although this relationship was not significant (P=0.174; Chi-square test; Table 4).

OVF was evaluated at one year after treatment (Table 5) and analysis included patients with menstrual status data. After 1 year, relevant data were available for 74 patients (75.6% of the study population). OVF was evident in 29 out of the 36 patients (80.6%) in the chemotherapy-alone group and in 17 out of 38 patients (44.7%) in the chemotherapy plus goserelin group (P=0.002). In the HR+ group, data were available for 45 patients (68.1%). OVF was evident in 20 out of the 24 patients (83.3%) in the chemotherapy-alone group and in 10 out of the 21 (47.6%) patients in the chemotherapy plus goserelin group (P=0.025). In the HR- group, data were available for 25 patients (78.1%). OVF was evident in 7 out of 10 patients (70.0%) in the chemotherapy-alone group and in 5 out of the 15 patients (33.3%) in the chemotherapy plus goserelin group (P=0.111).

### Disease Free Survival and Overall Survival

**Figure 1:**
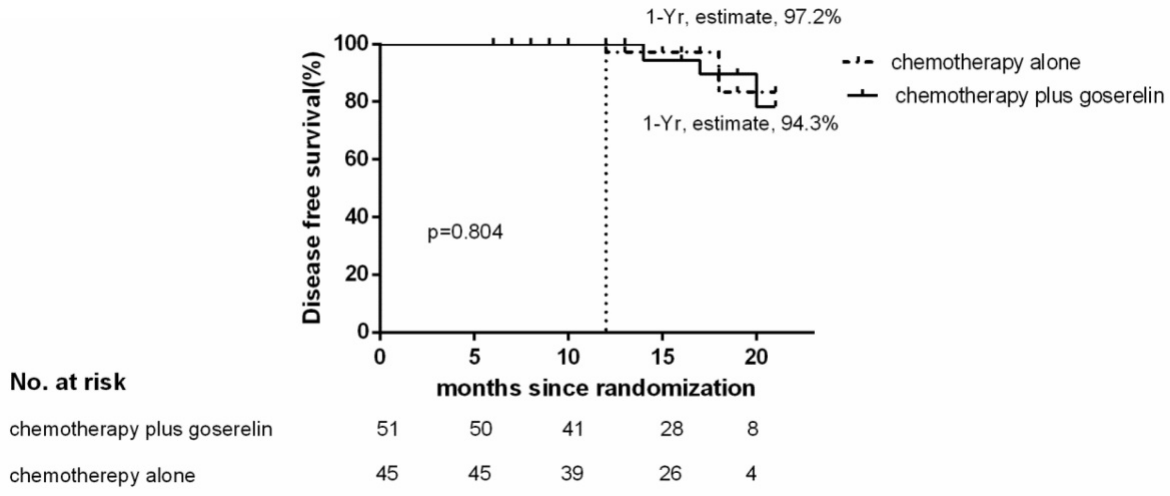
** Disease-free Survival.** The 1‑ year estimate of disease ‑ free survival is Kaplan-Meier estimates. There were 3 relapses or deaths in the chemotherapy-alone group and 4 in the chemotherapy-plus-goserelin group.

**Figure 2:**
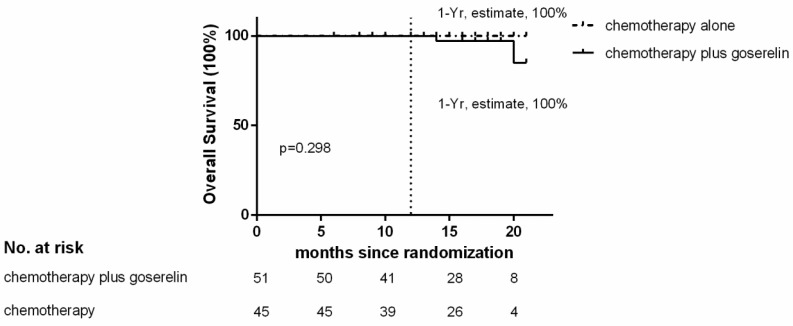
** Overall Survival.** The 1‑ year estimate of overall survival is Kaplan-Meier estimates. There were 2 deaths in the chemotherapy‑plus‑goserelin group and 0 in the chemotherapy-alone group.

Of the 96 patients analyzed, only 3 patients in the chemotherapy group, and 4 patients in the chemotherapy plus goserelin group experienced recurrence or died. The 1-year Kaplan-Meier estimate of the rate of disease free survival was 97.2% in the chemotherapy-alone group and 94.3% in the chemotherapy plus goserelin group (Figure 1). None of the patients in the chemotherapy-alone group died, although 2 died in the chemotherapy plus goserelin group. The 1-year Kaplan-Meier estimate of the rate of overall survival was 100.0% in the chemotherapy-alone group and 100.0% in the goserelin group (Figure 2). All 98 randomized patients showed no significant difference in terms of DFS and OS when compared between the chemotherapy-alone group and the chemotherapy plus goserelin group (P=0.804, P=0.298).

## Discussion

The latest clinical trial concluded that GnRHa, combined with tamoxifen or aromatase inhibitors, could improve disease free survival for hormone receptor positive (HR+) premenopausal high-risk breast cancer patients^20^. However, there is a lack of research addressing whether GnRHa can protect ovarian function during chemotherapy in patients with breast cancer. There are only few reliable randomized controlled studies and existing clinical findings are limited and inconclusive^21^. Several studies have indicated that GaRHa has no protective effect upon the ovary during chemotherapy; in these studies, it was notable that menstrual recovery ratio or menstrual recovery time were used as end points^22^-^24^. However, the PROMISE-GIM6^25^ study found that the proportion of patients with early menopause was significantly lower in a GnRHa group than in a control group. The POEMS^18^ study further found that ovarian failure was significantly lower in the GnRHa group than in the control group. Consistent with these two earlier studies, our present study concluded that OVF was significantly lower in the chemotherapy plus goserelin group compared to the chemotherapy-alone group.

For obstetricians and gynecologists, the most appropriate ovarian reserve screening tests to use in practice are basal follicle-stimulating hormone (FSH) plus estradiol levels or AMH levels. In particular, AMH is useful for this test as it remains stable throughout menstrual cycles and it can therefore be tested at any point 26. Our present study confirms and extends previous findings in that AMH declines rapidly 27, 28 and remains low during chemotherapy^27^. However, our present data are consistent with one previous study 29 in that AMH level was associated with age, and AMH levels in patients below 40 years were found to be higher than in patients over 40 years of age. Patients with higher baseline AMH are more likely to restore menstruation compared with lower AMH, which is consistent with previous study^30^. The reason may be that AMH is the most relevant hormone indicator of ovarian reserve capacity^27^. In addition, our study found that AMH levels decreased significantly during chemotherapy, and that there was no significant difference in AMH levels when compared between before chemotherapy and 1 year after chemotherapy. Furthermore, patients with higher AMH levels prior to chemotherapy had lower OVF when measured 1 year after chemotherapy. These results indicate that AMH can reflect the effect of chemotherapy upon ovarian function in real time and predict OVF after chemotherapy.

The current guidelines of the American Society of Clinical Oncology encourage patients of reproductive age to discuss fertility preservation if infertility is a potential risk of their proposed therapy^31^. More than one hundred babies have been born from frozen ovaries taken from cancer patients^32^, ^33^, but embryo cryopreservation involves time, cost, the use of sperm from a partner or a donor and a cycle of ovarian stimulation, which may limit assisted reproduction options for many young women who are receiving chemotherapy. GnRHa treatment, combined with chemotherapy, may be a more convenient option and can be used in combination with other fertility preservation techniques. Although this treatment has potential side effects, including the loss of bone density, it is anticipated that this treatment regimen can encourage the long-term preservation of ovarian function. Furthermore, for those patients who are not interested in fertility preservation, GnRHa may help avoid unwanted menopausal and osteopenia.

There has been far less research on the effects of combination chemotherapy including GnRHa on breast cancer survival. The POEMS^18^ study suggested that GnRHa combined chemotherapy can increase DFS and OS. However, our present study did not have positive results in terms of survival as in previous studies, possibly because of our short follow-up period.

Previous studies have concluded that GnRHa has an ovarian protective effect on HR-negative breast cancer patients during chemotherapy. Our study, despite our short follow-up period, showed that regardless of HR status, GnRHa has a protective effect on young breast cancer patients during chemotherapy. Our data provides further evidence for clinicians with regards to the use of GnRHa for HR+ breast cancer patients. In addition, AMH levels are significantly related to age and can reflect changes of ovarian function during chemotherapy and predict OVF after chemotherapy. AMH levels can provide doctors with additional information with which to draw up a strategy for fertility protection during chemotherapy.

In the future, we need more follow up time to prove that GnRHa can increase DFS for breast cancer patients and with more events, we can possibly further define the clinical use of GnRHa according to AMH levels during chemotherapy.

As a conclusion, Our study showed that GnRHa may have a protective effect on young breast cancer patients regardless of hormone receptor during chemotherapy. In addition, the level of AMH is significantly related to age, reflect changes of ovarian function during chemotherapy and predict ovarian failure after chemotherapy.

## Figures and Tables

**Table 1:** Baseline characteristics of patients, according to study group

Characteristic	All eligible patients		
	Overall	Chemo alone	Chemo plus Goserelin
	No (%)	No (%)	No (%)
Age			
Median(range)	39.0	40.0	37.0
<40 yr	53(55.2%)	21(46.7%)	32(62.7%)
>=40 yr	43(44.8%)	24(53.3%)	19(37.3%)
T stage			
T1	61(63.5%)	34(75.6%)	27(52.9%)
T2	30(31.3%)	9(20.0%)	21(41.2%)
T3	5(5.2%)	2(4.4%)	3(5.9%)
LN			
LN-	31(32.3%)	15(33.3%)	16(31.4%)
LN+	65(67.7%)	30(66.7%)	35(68.6%)
ER			
ER-	30(31.3%)	12(26.7%)	18(35.3%)
ER+	66(68.7%)	33(73.3%)	33(64.7%)
HER-2 status			
HER-2-	67(69.8%)	31(68.9%)	36(70.6%)
HER-2+	29(30.2%)	14(31.1%)	15(29.4%)

Chemo denotes chemotherapy.

**Table 2:** AMH, FSH, E2, LH, PRL, T level in different age groups before chemotherapy (Mann-Whitney test)

			All eligible patients				
	Overall		Chemo alone		Chemo plus Goserelin		P value
	Median level	No (%)	Median level	No (%)	Median level	No (%)	
AMH ng/ml	1.68		1.57		1.86		0.003
<40 yr	2.23	53(55.2)	1.68	21(46.7)	2.55	32(62.7)	0.453
>=40 yr	1.23	43(44.8)	1.33	24(53.3)	0.87	19(37.3)	0.002
FSH mIU/ml	6.30		6.52		5.97		0.119
<40 yr	5.91	53(55.2)	6.70	21(46.7)	4.98	32(62.7)	0.785
>=40 yr	6.82	43(44.8)	6.50	24(53.3)	8.30	19(37.3)	0.037
E2 pg/ml	50.25		56.00		43.00		0.271
<40 yr	48.80	53(55.2)	34.00	21(46.7)	53.50	32(62.7)	0.101
>=40 yr	53.00	43(44.8)	65.00	24(53.3)	43.00	19(37.3)	0.720
LH mIU/ml	5.07		4.63		5.59		0.218
<40 yr	4.90	53(55.2)	4.60	21(46.7)	5.14	32(62.7)	0.609
>=40 yr	5.27	43(44.8)	4.72	24(53.3)	5.87	19(37.3)	0.201
PRL ng/ml	19.61		20.07		17.92		0.067
<40 yr	23.04	53(55.2)	33.74	21(46.7)	21.19	32(62.7)	0.069
>=40 yr	17.74	43(44.8)	19.20	24(53.3)	17.15	19(37.3)	0.416
T ng/ml	0.44		0.44		0.42		0.455
<40 yr	0.44	53(55.2)	0.44	21(46.7)	0.51	32(62.7)	0.716
>=40 yr	0.41	43(44.8)	0.45	24(53.3)	0.40	19(37.3)	0.259

Chemo denotes chemotherapy.

**Table 3:** Change of AMH before and after chemotherapy (Wilcoxon Signed Ranks Test)

	Chemo plus Goserelin		Chemo alone	
	AMH	P value	AMH	P value
Before chemo	1.86		1.57	
1/2 cycles after chemo	0.12	0.000	0.10	0.000
6 months after chemo	0.04	0.000	0.03	0.000
1 year after chemotherapy	0.05	0.141	0.09	0.109

Chemo denotes chemotherapy.

**Table 4:** Association between OVF and age, AMH baseline level and chemotherapy treatment (Chi-square tests)

	Without OVF No (%)	With OVF No (%)	P value
Overall			0.002
Age<40	22(55%)	18(45%)	
Age>=40	6(17.6%)	28(82.4%)	
Over all AMH			0.018
Baseline<1.1ng/ml	4(17.4%)	19(82.6%)	
Baseline>1.1 ng/ml	24(49.0%)	25(51.0%)	
Endocrine therapy			0.174
Without	10(40%)	15(60%)	
With	11(22.9%)	37(77.1%)	
GnRHa			0.002
Without	7(19.4%)	29(80.6%)	
With	21(55.3%)	17(44.7%)	

Chemo denotes chemotherapy.

**Table 5:** Association between GnRHa and OVF (Chi-square tests)

	Chemo alone		Chemo plus Goserelin		P value
	Without OVF	With OVF	Without OVF	With OVF	
Over all	7(19.4%)	29(80.6%)	21(55.3%)	17(44.7%)	0.002
HR-	3(30%)	7(70%)	10(66.7%)	5(33.3%)	0.111
HR+	4(16.0%)	21(84.0%)	11(47.8%)	12(52.0%)	0.029

Chemo denotes chemotherapy.
